# Perceptions on hypertension among migrants in Delhi, India: a qualitative study

**DOI:** 10.1186/1471-2458-9-267

**Published:** 2009-07-28

**Authors:** Yadlapalli S Kusuma

**Affiliations:** 1 Centre for Community Medicine, All India Institute of Medical Sciences, New Delhi – 110 029, India

## Abstract

**Background:**

The developing countries are experiencing epidemiological transition and hypertension has emerged as a major threat to health in these countries. Understanding people's perceptions is important for any prevention and control activities and lay explanatory models (EMs) provide an opportunity to gain insights into the people's perceptions. This qualitative study is taken up with an objective of understanding EMs of neo- and settled-migrants regarding hypertension.

**Methods:**

Qualitative methods with grounded theory approach were used to elicit EMs of hypertension held by neo- and settled-migrants. In-depth interviews with key-informants and focus group discussions with community members were conducted. The data were subjected to thorough reading and analysed by segregating the text under different themes.

**Results:**

Hypertension has been perceived as a common and serious problem in the community. Lay conceptions have identified hypertension as symptomatic with ambiguity over perceived symptoms. City life has been perceived as a major predisposing factor for developing hypertension. City life has been corroborated with pollution and adulteration of food, stress, high fat diet along with physical inactivity and certain attitudes. The concepts of hypertension were interconnected and linked to their day-to-day living in the city. Inadequacy of awareness has been acknowledged and there was a felt need for awareness campaigns and screening programmes in the community. The EMs of hypertension among the neo- and settled-migrants and men and women were broadly similar. However, there were slight variations by gender and migration status in the perceived pathways to hypertension.

**Conclusion:**

Hypertension has been perceived as a common and serious problem in the community. Hypertension has been perceived as symptomatic; however, ambiguity prevails over perceived symptoms. Awareness and knowledge about hypertension and its consequences are inadequate in these communities. The felt need for awareness campaigns and mass screening programmes has emerged from the community and it provides enabling environment to successfully carry out public health interventions, by addressing the existing gaps, for prevention and control of hypertension and other cardiovascular diseases.

## Background

Hypertension has become a significant problem and contributor to other cardiovascular diseases (CVD) in many developing countries experiencing epidemiological transition.[1,2] Asian Indians have increased risk of coronary events, and reasons for this increased risk are thought to be genetic but are yet unclear.[3] In addition, there is a strong correlation between changing lifestyle factors and increase in hypertension in India.[4] Moreover, recent research indicated that low socio-economic communities are not exempted from the risk of hypertension.[5,6] The socio-economically disadvantaged communities like migrants in large cities are vulnerable to hypertension. [7-9] In this context, there is an urgent need for prevention and control of hypertension.[10,11] It is not known how the community perceives and views the problem of hypertension. Hence, understanding the lay beliefs and perceptions are important as prevention and control of chronic conditions such as hypertension requires life long adoption of healthy lifestyles. Also, they are important in identifying and bridging the gap between the people and health care providers. Explanatory models (EMs) through qualitative enquiry are an essential tool in unravelling the people's perceptions. According Kleinman, health care providers and patients have different EMs of sickness and treatment, including explanations for the aetiology of the condition, the timing and onset of symptoms, the patho-physiological processes involved, the natural course and severity, and appropriate treatments [12] and EMs are culturally constructed explanations for a specific illness and its treatment.[13] The aim of the study was to understand EMs held by socioeconomically disadvantaged neo- and settled-migrants in Delhi regarding hypertension by using a grounded theory approach.

## Demographic context

Delhi, the national capital of India is experiencing greater influx of migrants due to rapid developmental activities.[14] In this study, two groups of migrants namely, neo-migrants (defined as those who have migrated to the city of Delhi from rural villages within last two years and this being their first migration) and settled-migrants (those who have migrated and residing in Delhi at least since 10 years) were considered. Both the groups of migrants are mainly from northern Indian states, and their distribution in terms of origin and ethnicity was similar and comparable. However, settled-migrants are relatively better in terms of education, income and acquaintance with the city compared to the neo-migrants. While many of the settled-migrants live in their own houses in resettlement colonies, the neo-migrants live in squatter slums with dilapidated houses. People migrate to urban areas with the expectation of better earnings and quality of life. However, low socio-economic conditions along with the new environment makes these migrant populations vulnerable. They have less access to basic amenities, education, and health, and the integration of migrants with the local population is impeded due to their vulnerability. The prevalence of hypertension among neo- and settled-migrants was 16% and 20% respectively.[7]

## Methods

This study used a grounded approach [15,16] to develop an understanding about how disadvantaged migrants in Delhi view hypertension. Data were collected through in-depth interviews with key-informants and focus group discussions with community members. The purpose of key informant interviews is to learn about peoples views on the topic of interest, to learn their terminology and judgements and to understand their perceptions and experiences.[17] Focus groups can yield more and richer information than individual interviews with the same participants. [18,19] They provide broader insights on and better understanding of how people perceive a specific problem. The focus groups can paint a picture of what is socially acceptable in a community rather than what is really occurring or believed, while in-depth interviews are more appropriate method in exploring the complex beliefs of individuals.[20] Hence, I used a combination of these two qualitative methods, which is appropriate for the present study.

### Study Participants

Key-informants were both men and women of neo- and settled-migrants. They have close interactions with the group and have good reputation. They are good observers in the sense that they were aware of the ongoing activities and changes in the community and were involved in various socio-cultural, religious, recreational activities. Initially few key-informants were identified and subsequently some more key-informants were identified by using snowballing technique.[21] Thus, a total of 14 key informants were selected (Table 1). All women key informants were housewives. All neo-migrant men were daily wage labourers, while the settled-migrant men key informants were involved in skilled works or small business. One neo- and two settled-migrant women did not receive any formal education, while the rest of the settled-migrant women participants were educated with primary school education. All men except two neo-migrants received formal school education.

**Table 1:** Details of key-informants

	**Settled-migrants**	**Neo-migrants**	**Total**
Male	3	3	6
Female	5	3	8
Age Range	38–50 years	22–40 years	38–50 years
Mean age ± SD	46.5 ± 5.13 years	34.5 ± 4.81 years	41.4 ± 7.8 years

Three focus groups discussions (FGDs) were conducted with community members following standard guidelines.[20,22] A focus group of 6–10 people are usually recommended,[23] and groups with more than eight are difficult to control.[20] The recruitment of participants was purposive. In purposive sampling, researchers chose study sites or informants to represent the range of variation in those characteristics that seem to be meaningful for the topic under study. In this situation, a small number of specially chosen informants can yield valid and generalizeable information.[24] Thus, it was decided to constitute focus groups based on their migration status and gender. The research team (consisted of the author, who is the principal investigator of the project, and two project assistants) was familiar with the community and developed good rapport during the preceding survey and, thus were able to identify people who are information rich. Initially 8–10 participants were invited to participate in the discussion. However finally, 6–8 participants turned up for discussion (Table 2). All women participants were (FGD1 and FGD3) were housewives. Three of the neo-migrant women (FGD3) and two of settled-migrant women (FGD1) did not receive any formal education while the rest of the participants received education up to primary level. All men participants (FGD2) received formal education up to 12^th ^and one attained graduate education. They were involved in either salaried jobs, skilled works or have small business.

**Table 2:** Details of the focus groups

	**FGD 1**	**FGD 2**	**FGD 3**
Migration status	Settled-migrants	Settled-migrants	Neo-migrants
Gender	Female	Male	Female
Number of participants	6	8	6
Age range	25 – 50 years	30 – 50 years	25 – 40 years
Mean age ± SD	35.8 ± 8.6	39.4 ± 7.76	29.5 ± 3.39

### Study Instruments

The topic guides were built upon the previous studies on hypertension and earlier work on perceptions on illness.[13,25] Topic guides were developed in English and translated to Hindi, the local language of Delhi. They were pre-tested for their relevance, suitability and ease to carry out in the community.

For in-depth interviews, the set of questions in the topic guide was pre-tested among some individuals (who have hailed from socially disadvantaged areas, however presently not living in those areas) working in the author's institute. The final main question route for in-depth interviews was: Whether hypertension is a serious problem in their community? What are the causes of hypertension? Were your community members aware of hypertension? What are the consequences of unawareness? What do you suggest to prevent hypertension? Several probes were made taking the leads from the narrations of the participant. The common probes were: how living in cities is responsible for getting hypertension? Elaborate on the lifestyle factors you were telling about, etc. Finally, every participant was asked, would he/she likes to tell any thing more about hypertension?

Topic guide for the focus group was discussed amongst the research team and pretested among a group of women, who have hailed from socially disadvantaged areas, but presently not living in the study areas. The topic guide for focus groups included the following main question line: What are the diseases that are common in your area? Is hypertension a problem in your community? What are the reasons for hypertension? What type of people get hypertension? Are all those who were expected to have hypertension were aware of the problem? What are the consequences of unawareness? Can we discuss anything more about hypertension?

### Study Procedures

Standard methods were followed while conducting key informant interviews.[26,27] All in-depth interviews were conducted in the participants' homes. All the interviews were audio-taped. The Duration of the in-depth interviews ranged from 40 to 75 minutes. At the end of the day, the researchers discussed the highlights of each interview and important aspects of the narrations were noted down. These notes were thoroughly examined for new information and repetitions. Selection of key informants continued till saturation of data has occurred.

Focus groups discussions (FGDs) were conducted with community members following standard guidelines.[20,22] The author moderated the sessions, and project assistant took care of operating the tape recorder and taking notes of the discussion. The other project assistant watched and took notes of participants' actions, gestures and emotions that could not be captured on audio tape. The discussions were conducted in Hindi and the entire discussions were audio-taped. The focus group sessions followed the following pattern: i) welcome and self-introduction, ii) explanation of the study and consent taking iii) discussion proper iv) de-briefing of the discussion, v) allowing participants, if they have anything to say. and vi) appreciation for participation. Each session took 1 1/2 to 2 hrs. The research team discussed on what had transpired and the observer's field notes on impressions and observations.

### Ethical considerations and Informed consent

The project protocol has been approved by the Ethics Committee of the All India Institute of Medical Sciences, New Delhi, India. All participants were informed individually about the study objectives and all the participant gave their consent for interviewing and recording prior to participation. No incentives were given to the participants.

### Data management and analysis

All key informant interviews and focus group discussions were transcribed into Hindi while listening to the audio-tapes and simultaneously checking with the notes taken during interview/discussion. The entire transcriptions were read while listening to the audio-tape for accuracy of transcription. All these Hindi transcripts were translated into English by one project assistant. However, some portions of the Hindi transcripts were translated separately, by two project assistants, and some were back translated to check linguistic reliability and correctness in translation. The data were entered into a word processor.

The data of interviews were read thoroughly several times. Later the data were reorganized under different themes based on the topic guide. Under each theme, several codes were identified and relevant quotes were kept under each code. Inferences were drawn collectively by careful reading of the text under each code. These data were read and re-read to check the appropriateness of the inferences drawn.

Each focus group discussion was read separately. Again in each discussion, the discussion for a theme was read at a time, and the highlights of that part of discussion were made as notes by simultaneously copying and pasting the relevant quotes under this notes. This is followed for each theme in the discussion. Thus each focus group discussion was reorganized under the identified themes. Then, this reorganized data (quotes) were read and re-read and the inferences were drawn. This is followed for each of the three focus groups separately, and final inferences were drawn by synthesizing inferences from all these groups.

Thus, analyses were carried out separately for in-depth interviews and focus group discussions. However, the inferences drawn from both types of data were utilized while presenting the results. The coding was done by the author only. However, the inferences drawn from the analyses for in-depth interviews as well as for focus groups were checked through discussions with another anthropologist (who is not involved in the project), who read the data independently and gave the feedback.

## Results

The results based on the inferences drawn from in-depth interviews and focus groups are presented under four major headings namely, (i) the problem of hypertension in the community, (ii) causes of hypertension, (iii) awareness of hypertension status and (iv) suggestions for preventing hypertension.

### The problem of hypertension in the community

Hypertension is called as '*BP' *(blood pressure) in these communities. The participants explained that if a person 'has BP' means that she/he is hypertensive. They were also aware of low blood pressure and specifically mentioned that 'BP is low' or 'low BP'. A term for blood pressure exists in Hindi (*rakta chaap*), however it is not in popular usage. Thus, hypertension is referred to as BP and researchers also used 'BP' to refer to hypertension during interviews and discussions. Hypertension was perceived as a serious and common problem in their community. A 50 years old settled-migrant female key informant expressed that *"Yes. BP is a common problem these days. It is mainly due to the growing tensions....this problem is being faced in every household. Earlier BP was associated with only rich people but now-a-days BP along with heart problems has become very common among us"*. During focus group discussions, hypertension was mentioned as a common and serious problem in their community along with other illnesses such tuberculosis, fevers, diabetes, and other respiratory illnesses. During focus group of settled-migrant men, one participant (30 year old male) expressed that hypertension is not a very common problem in their community and a discussion amongst the participants took place over this issue. However, the group arrived consensus that hypertension is a problem among their community. All participants perceived that hypertension was not a problem in the rural, which was their previous habitation.

### Causes of hypertension

**Figure 1:**
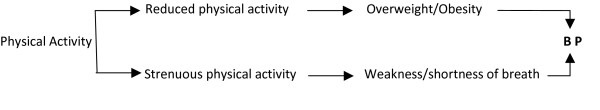
**Perceived pathways of physical activity to BP (local term for hypertension)**.

The present EMs identified several causes of hypertension such as old and middle age, dietary habits, obesity, physical activity, tensions, etc as responsible for increasing prevalence of hypertension. It was uniformly felt that hypertension would not affect children, as they have neither heard nor seen a child known to be hypertensive. Other perceived risk factors were female gender and urban living. Diet rich in fats and salt as well as lack of adequate and good diet were linked to hypertension. During focus group of settled-migrant women, it was expressed that *"One of the reasons is the eating habits of people; now-a-days people are consuming more of fats... All these lead to BP" *During focus group of neo-migrant women *"Poor and weak people get blood pressure. Blood becomes thin and hence it increases in volume. As the volume of diluted blood is more, it flows faster resulting in blood pressure"*. Physical inactivity as well as strenuous physical activity were linked with hypertension (Figure 1). Again, diet and physical activity were linked to hypertension through different pathways, however, stress was perceived as a major cause of hypertension uniformly by all the participants and the identified sources of tensions were common among both the groups. The sources of tensions include unemployment, rising prices, insecure living, daily hassles in the city life (such as basic amenities like water supply, space, travel, time and work related issues), disharmony in the community, behaviour of the adolescent children and men, problems related to children's education and health, responsibilities like daughters' marriage (which involves dowry), eagerness to earn and grow more, corruption, urban attitudes, etc. A 38 years old settled-migrant male key informant mentioned *"tensions... family members who are working... they are tensed of their daily routine, accidents happening on roads, tensions of office and other problems"*.

All the data from key-informant interviews and focus groups revealed convergence of issues regarding various aspects of hypertension. However, there were few differences in the perceptions of neo- and settled-migrants as well as men and women. While the EMs of the settled-migrants mainly centred on the changed dietary habits, physical inactivity and stress; the explanations of the neo-migrants highlighted stress followed by dietary factors and physical activity as precursors of hypertension. Some perceived that women are more prone for hypertension, however, explanations offered for this perception varied between women and men. Women participants perceived that women were at high risk of developing hypertension as tensions are a routine in woman's life. The men participants expressed that women tend to become overweight/obese after marriage and child bearing and thus are more prone to hypertension. Neo-migrant women's group attributed the source of tensions to the behaviour of husbands and egoistic nature of children. A typical comment during focus group of neo-migrant women "*Wives are being troubled by their husbands. Sons do not listen to their parents, roam around, do not study; whereas girls make relationships and have affairs. Some husbands fight with their wives after drinking. All these things lead to BP"*.

**Figure 2:**
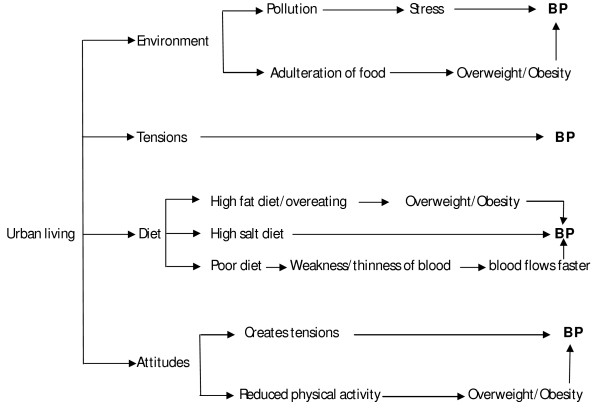
**Perceived pathways of urban living to BP (local term for hypertension)**.

*Urban living as a cause of hypertension *– All the participants felt that living in the city was associated with hypertension. The major factors identified were (i) pollution and adulteration of food, (ii) tensions, (iii) dietary factors and (iv) attitudes of people. All these factors were interconnected and linked to their day-to-day life. Figure 2 depicts the perceived pathways of urban living leading to hypertension as follows. (i) The pathway of pollution to hypertension was explained through the route of stress to hypertension while the adulteration of food through obesity to hypertension. During focus group discussion with settled migrant men, it was expressed *"All this is due to various adulterations in oils that we use for cooking, it makes one overweight and get BP"*. *(ii) *Tensions were perceived as another major cause of hypertension. A 50 years old settled-migrant female key informant said "*..... people have lot of tensions in their lives. There is unemployment, price rises. People cannot eat properly due to rising prices, this leads to BP because everything is so expensive how would poor people live and survive..." *(iii) Diet rich in fats and salt along with reduced physical activity were perceived as other causes of hypertension. Over eating, preferences for fried, junk food to traditional food, and eating adulterated food were linked to urban living. A 25 years old neo-migrant female key informant expressed, *"Chances *(of getting hypertension)*are higher as people in cities do not want to follow any restrictions; they eat whatever comes their way and most of the times they over eat. In villages the women work whole day whereas in cities women sit at home and eat all the time..."*. (iv) Attitudes of people in cities were cited as predisposing factors for developing hypertension. These include physical inactivity (in terms of preference to motor vehicles over walking, sitting at home all the time) and quarrelsomeness (in terms of lack of tolerance and tendency to quarrel over small issues). During the focus group of settled-migrant men, *"The lifestyle in Delhi is such that even if one has to go half-a-kilometre away, one would go on bikes and all whereas in villages people do not mind to walk five kilometres at a stretch. That makes the difference"*. A 33 years old neo-migrant male key informant said *"In village, people are not short-tempered, whereas in cities, people get irritated and angry even over very small issues...."*.

### Awareness of hypertension status

All participants acknowledged that awareness of hypertension status was not adequate in their communities. Some expressed that several people were not aware that they were hypertensive, while others indicated that some hypertensives knew about their hypertension status. However, all the focus groups and key-informants opined that it is difficult for the individual to know his/her hypertension status, unless checked by health care personnel. They indicated that due to individual's lack of knowledge regarding various symptoms of hypertension, people were unaware of their blood pressure status.

The participants perceived hypertension as symptomatic, however, they expressed that there was ambiguity regarding linking some symptoms only to hypertension and expressed that these symptoms were associated with other illnesses also. The participants acknowledged the consequences of unawareness of hypertension that hypertension may lead to several other cardiovascular diseases and haemorrhagic stroke was perceived as the most serious and fatal consequence of hypertension. The settled-migrant participants expressed that unawareness takes a greater toll on the life of the individual. In focus group of settled-migrant women, *"No body knows what will happen with them if BP is not treated. For instance my husband didn't have much of any problem but he used to have shortness of breath, but we never imagined that for this type of problem will come out with a heart problem*. The neo-migrants women participants expressed that they can't clearly mention the consequences of unawareness. Thus, unawareness of hypertension status and poor knowledge were prevalent in the community.

### Suggestions for preventing hypertension

Participants emphasized on the dietary aspects in terms of low salt diet, vegetarian diet, avoiding fried and commercially available foods and exercises by means of walking and yoga. Several participants opined that bringing dietary changes is important however that it is difficult to resist certain foods for ever and hard to sustain the lifestyle changes. A 33 years old neo-migrant male key informant suggested, *"To prevent BP, we should consume more of green leafy vegetables, take fresh air, eat healthy and light food and it is very important to eat, sleep and wake up in time"*. It was further opined that though lessening tensions can prevent hypertension, tensions are unavoidable in their lives and each and every household will have problems that cause tensions. They also suggested that though it is impossible to completely avoid domestic tensions, one should try to stay cool, calm and control one's anger.

#### Awareness helps preventing hypertension

The key-informants and focus groups suggested and felt the need for blood pressure screening and opined that awareness campaigns regarding the problem of hypertension, its symptoms and risk factors would help people to protect themselves and make them seeking medical care. They informed that so far no campaign regarding hypertension had taken place in the community. The settled-migrant men's focus group expressed that *"If people have full knowledge, then it's possible to prevent BP. Then only people can try to control, other wise, consequences can be fatal"*. Similar expectations arose from neo-migrants also. A 40 years old neo-migrant male key informant opined *"Government should bring about awareness among labour class who are always neglected. We have always seen banners and advertisements for TB and AIDS but never seen such things for BP. Campaigning will bring awareness"*.

Thus, issues emerged in in-depth interviews and focus group discussions were convergent and similar. However, in-depth interviews mainly provided insights into the intra- and inter-individual level factors in causation and prevention of hypertension. While lack of self-efficacy to bring life-style changes mainly emerged during focus group discussions. Also, the focus groups mainly yielded perceptions regarding environmental and contextual factors in the causation of hypertension and expectations of people took the central stage.

## Discussion

Before considering the implications of the study, it is important to note the limitations in this study, as usual to this type research. The preconceptions of the researchers, based on previous studies, such as proneness of urban people specifically with obesity, and stressful life; lack of awareness regarding hypertension, might have influenced the development of topic guides to some extent. Focus groups have limited value in exploring complex beliefs; however, in-depth interviews, which are more appropriate for this purpose, were also employed. In focus groups, some participants are very vocal while some are inactive, however, these issues were managed by encouraging inactive participants and requesting the vocal participants to let the other participants express first. Only three focus groups were conducted due to limited resources. The author moderated the discussions and this may lead to some bias. Two project assistants were employed with the author only during data collection, transcription and translation of data and hence, analysis was carried out by the author. However, the data were discussed after the completion of interviews/focus group discussions amongst the research team.

This study is first of its kind from India highlighting the perceptions in the community's perspectives. The purpose of this study was to recognize the EMs of the problem, causes, and consequences of hypertension in neo- and settled-migrants in Delhi. The EMs of both the migrant groups were similar in many respects. The explanations revealed that hypertension was perceived as a common and serious problem in the community. While the medical model of hypertension mainly centred around the physical aspects, the lay perspective centred on the social aspects such as unemployment, raising prices, insecure livelihood, disharmony in the community, and other urban related issues. Beune et al.[28] also found that the EMs of hypertension in patients from different migrant groups differed from the common medical perspective. Dela Cruz and Galang [29] found that the EMs of Filipino Americans correspond to the biomedical model in relation to causes, consequences and treatment. In the present study, diet, physical inactivity, stress and urban living were identified as major causes of hypertension and these were interconnected and linked to day-to-day life. Stress was perceived as an important causal factor in the present EMs and was consistent with other studies.[25,28,30-33]

The present EMs had a physiological dimension as demonstrated by the perceived pathways of physical activity (Figure 1) and urban living (Figure 2) leading to hypertension. Though hypertension is said to be asymptomatic, the present EMs considered hypertension as symptomatic. However, blood pressure check-up has been perceived as the only means of identifying one's hypertension status as ambiguity prevails over perceived symptoms.

The present EMs also revealed that perceived susceptibility and seriousness were low in the community. Low levels of risk perception were reported by other studies.[29,34] The participants highlighted several behavioural risk factors such as unhealthy diet and physical inactivity, however, no mention was made regarding tobacco use and alcohol consumption despite the fact that these were far more widely consumed by the socio-economically disadvantaged communities.[10]

There was felt need for awareness campaigns and mass-screening programmes for hypertension. Awareness regarding the risk factors should be made accessible to the public through various means such as Information, Education and Communication (IEC) campaigns, along with provision of primary health services and proper referral. The primary health care system in India has put more emphasis on IEC and preventive activities related to infectious diseases and family planning and only meagre attempts have been made to prevent CVDs and their risk factors including hypertension. However, recently, the government of India has launched a programme for prevention and control of diabetes, cardiovascular diseases and stroke, and IEC is part of it.[10,35] These activities are yet to reach the communities. Since hypertension is an important public-health challenge worldwide; prevention, detection, treatment, and control of this condition should receive high priority.[36] Effective community education programmes are vital to increase public knowledge and awareness of hypertension and related cardiovascular diseases. Powers et al. [34] while studying the perceived risk emphasized the need for better patient education on the risks associated with hypertension. Vergara et al.[37] has pointed out that, patients' perceptions and awareness of health-related issues specific to a particular medical condition play an important role in the management and outcome. The present study along with other studies [29,34] felt the need for cardiovascular health promotion strategies, and further emphasizes the importance of designing culturally sensitive and congruent health education and promotion activities.

## Conclusion

Hypertension has been perceived as a common and serious problem in the community. The lay EMs differs from the bio-medical model. The study concludes that awareness and knowledge about hypertension and its consequences are inadequate in these communities. Inaccurate public understanding of hypertension and its consequences contributes to low levels of perceived susceptibility, seriousness and self-efficacy to bring lifestyle changes in preventing hypertension. There was felt need for awareness campaigns along with screening for hypertension and this may help implementing the prevention and control activities by addressing the above gaps by the health system.

### Implications

Qualitative studies of this kind are important not only to get an insight into the lay beliefs, but also have implications for providing community specific health care, particularly for those who are marginalized for the reasons of social and economic status, migratory status, ethnicity, etc. In the wake of rising burden of CVDs in developing countries, national level preventive and control activities need this type of inputs. This information is helpful in identifying existing gaps in knowledge and awareness and thereby useful in designing health promotional activities relevant to the communities, specifically to migrant communities existing in urban areas in India and elsewhere. The inadequate knowledge on causation, prevention and un-sustained life-style changes should be addressed during these activities. Also, screening and counselling should be made accessible for communities with an endeavour to foster positive beliefs and self-reliance regarding life-style changes.

## Competing interests

The author declares that they have no competing interests.

## Authors' contributions

YSK designed the study, collected and analysed the data and wrote the paper. YSK is guarantor.

## Pre-publication history

The pre-publication history for this paper can be accessed here:


